# Effect of *Rosa damascena* Extract on Rat Model Alzheimer's Disease: A Histopathological, Behavioral, Enzyme Activities, and Oxidative Stress Study

**DOI:** 10.1155/2023/4926151

**Published:** 2023-04-10

**Authors:** Leila Beigom Hejaziyan, Seyed Mohammad Hosseini, Ali Taravati, Mohammad Asadi, Mahyar Bakhshi, Pedram Moshaei Nezhad, Mohammad Gol, Mobina Mououdi

**Affiliations:** ^1^Cellular and Molecular Biology Research Center (CMBRC), Babol University of Medical Sciences, Babol, Iran; ^2^Department of Human Anatomy, Faculty of Medicine, Babol University of Medical Sciences, Babol, Iran; ^3^Department of Pathology, Babol Branch, Islamic Azad University, Babol, Iran; ^4^Department of Molecular and Cell Biology, Faculty of Basic Sciences, University of Mazandaran, Babolsar, Iran

## Abstract

The purpose of the current study is to investigate the effect of aquatic *Rosa damascena* extract against the oxidative damage induced by aluminum chloride intoxication in Alzheimer's model of Wister rats. Rats were divided randomly into seven groups (*n* = 10). Control group received no treatment, sham group received distilled water orally, aluminum group (AL) was administered AlCl_3_ (100 mg/kg) orally, extract 1 and 2 groups were treated with only aqueous *R. damascena* extract (DRE) (500 and 1000 mg/kg), and treatment 1 and 2 groups received aqueous *R. damascena* extract (500 and 1000 mg/kg) and AlCl_3_ (100 mg/kg) orally. The brain tissues were sampled for histopathological examination, and biochemical analysis was conducted for estimating the enzyme activities of acetylcholinesterase and catalase (CAT), the levels of GSH and MDA, and ferric reducing antioxidant power. According to the results of behavioral tests, AL administration showed a reduction in spatial memory and remarkably increased the time needed for reaching the invisible platform. The administration of Al-induced oxidative stress and an increase of the enzyme activity of AChE. Al administration increased AChE level from 1.176 ± 0.173 to 3.62 ± 0.348, which was a significant rise. However, treating with the extract at the dose of 1000 mg/kg downregulated it to 1.56 ± 0.303. Administration of the *R. damascene* extract caused an increased level of catalase and glutathione levels in treatment groups, attenuated MDA level, and regulated AChE activity. Our results illustrate that administration of *R. damascene* extract has a protective effect against the oxidative damage induced by AlCl_3_ intoxication in Alzheimer's model.

## 1. Introduction

Aluminum (Al) is an abundant metal in our environment used widely by humans, which makes toxicity particularly relevant to human health. This metal is widely identified as a neurotoxin. A relationship exists between exposure to aluminum and neurodegenerative diseases, such as Parkinsonism dementia, amyotrophic lateral sclerosis, and Alzheimer's disease (AD). Exposure to high levels of Al leads to neurofibrillary degeneration, and higher Al concentration is associated with degeneration, oxidative stress, neuronal apoptosis, and neurotransmission alterations in AD [1, 2].

This metal is known to speed up extracellular *β* amyloid generation and aggregation. It acts as a cholinotoxin and causes alterations in the cholinergic activity, a key event in the neurochemistry of AD [3]. Also, it is known to speed up extracellular *β* amyloid generation and aggregation. Moreover, it can act as a cholinotoxin and causes alterations in the cholinergic activity, a key event in the neurochemistry of AD [3]. However, many medicines, toothpaste, food additives, and even water contain aluminum [4]. The most common form of human exposure to aluminum is through the gastrointestinal tract with a rate of absorption, of about 0.2% [5].

Alzheimer's disease is a common form of dementia in aged people that is characterized by a gradual loss of neuronal function, memory loss, and a decline in language skills [6, 7]. The AD is defined by the following two pathological features: b-amyloid protein (A) deposition and tau hyperphosphorylation. Age, gender, genetic factors, and some environmental factors such as a history of head trauma, diabetes, hypothyroidism, increased cholesterol, and accidental use of aluminum and zinc, as well as lifestyle may contribute to the risk of the AD [8]. High levels of Al have been reported in the brain of patients with AD relative to controls [5]. In this regard, the correlation between aluminum and AD appears stronger than that of other neurological disorders [9]. Oxidative stress poses a significant risk to AD. It is believed that redox abnormality is involved in the neurodegenerative process leading to the impairment mediated by reactive oxygen species and reactive nitrogen species in the AD brain. Besides, some studies reported that the levels of oxidative markers of biomolecules including nucleic acids, lipids, proteins, carbohydrates, and antioxidant enzymes changed. It is believed that natural antioxidants are neuron protectants [10].

Numerous traditional and herbal medicinal plants are being employed to treat neurological disorders such as AD, which act as antioxidants, anti-inflammatories, and cholinesterase and *β*-secretase inhibitors. These natural compounds also prevent the accumulation of amyloid beta and its fibril formation [11].

*Rosa damascena*, known as Damask Rose, belongs to the family of *Crassulaceae* and grows in Northern Asia and the mountains of Central Europe [12]. For several centuries, this plant has been used as an advantageous and effective treatment for a wide range of diseases and in folk medicine for treating many diseases such as anti-inflammatory treatments [13], menstrual bleeding, digestive problems [14], strengthening the heart and chest [15], and abdominal pain. Moreover, *Rosa damascena* is widely used in Iran's traditional medicine in various forms, such as extract, powder, and essential oil. In addition, this plant has multiple active ingredients, which are massively investigated in numerous studies for their bioactive effects, such as anticancer and antioxidant properties [16]. This important ornamental plant has long been applied for industrial, physiological, and medicinal purposes [17]. The physiological functions of Damask Rose may be somewhat related to the plenty of anthocyanidins and flavonoids. Anthocyanidins and flavonoids possess a broad spectrum of biological operations and play an important role in human health as astringents, anti-inflammatory agents, antioxidants, and free radical scavengers. The treatment properties of this plant may include stimulating the nervous system, reducing depression and anxiety, and improving performance [18–20]. There are essential compounds of flavonoid glycoside in this plant, which inhibit the activity of the AChE enzyme. Therefore, due to the presence of such compounds, the plant will be able to control the disturbances caused by AChE [21].

The present study aims at providing a behavioral, biochemical, and histopathologic evaluation of the effect of aquatic *R. damascene* extract against the oxidative damage induced by aluminum chloride intoxication in Alzheimer's model of Wistar rats.

## 2. Materials and Methods

### 2.1. Animals

Adult female albino Wistar rats (approximately 8–10 weeks old, 200–250 g) [22, 23] were purchased from the Pasteur Institute of Iran, North Research Center (Amol, Iran) [24]. Rats were kept in an insulated room with a 12-h light/dark cycle at an ambient temperature of 22 ± 3°C and relative humidity of 52 ± 5% and were fed adequately and appropriately [25, 26]. Experimental methods were approved by the ethics committee of Babol University of Medical Sciences with the approval ID *Mubabol.rec.1394.24*.

### 2.2. Chemicals

All chemical compounds for biochemical examination were procured from Sigma (Sigma–Aldrich GmbH, Steinheim, Germany).

### 2.3. Plant Extract Preparation

Fresh petals of *R. damascena* were collected from Kashan 34.0258°N, 51.0540°E, Iran. A voucher specimen of the plant (HUMZ 8115) was deposited in the herbarium center.

20 grams of fresh petals were placed in a 200 mL conical flask, and 100 mL of water was added. The Erlenmeyer flask was covered and kept in a reciprocating shaker for 24 hours for persistent agitation for thorough mixing and complete elucidation of active materials to dissolve in water. Then, the extract was filtered and the water from the extract was removed by using a rotary vacuum evaporator. Eventually, the remainders were gathered and used [27].

### 2.4. Experimental Design

Prior to starting the study, rats were divided randomly into the following seven groups (*n* = 10): control group: received no treatment; sham group: received distilled water orally for ensuring they receive the same administration stress as the treatment groups; group AL: administered AlCl_3_ (100 mg/kg daily) (dissolved in distilled water) orally for four weeks [28]. Groups DRE 500 and DRE 1000 were treated with aqueous *R. damascena* extract (500 and 1000 mg/kg) [29, 30] daily for eight weeks orally and groups AL + DRE 500 and AL + DRE 1000 received aqueous *R. damascena* extract (500 and 1000 mg/kg) as gavage daily for eight weeks and AlCl_3_ (100 mg/kg) orally daily for the last four consecutive weeks.

### 2.5. Separation and Identification of the Constituents of the Extract

The chemical content of the extract was analyzed by a gas chromatograph mass spectrophotometer (GC-MS) system (Agilent Technologies 7890A). GC/MS analyses were performed using the Agilent Technologies device with a *μ*m × 30 m column (HP-5MS 250 and a thickness of 0.25 *μ*m). The column temperature was kept at 50°C for 2 min and stored at 200°C for 5 min and 280°C for 12 min. Helium gas flow rate was used as carrier gas at a speed of 1 ml/min at 70 eV. The constituents of the extract were determined to calculate the index of inhibition of compounds and to compare their mass spectra with library resources (e.g., library and device) and resources such as the Eight Pick Index [31].

### 2.6. Behavioral Tests

#### 2.6.1. Single-Trial Passive Avoidance Test

It was administered using the shuttle box (BPT Co., Tehran). This device is equipped with two dark and bright chambers of equal size connected through a small and central guillotine door. Electric shocks were applied through a metal lattice floor when the rat was in the dark room. All rats were adapted with a device for the first two days in the dark chamber for 5 min. On the third day, they entered the bright chamber and after staying there for 2 min, the door was opened. Due to the lack of desire for light, the rats entered the dark chamber and the door was closed. Afterward, an electric shock was given to the rats at a rate of 2 s and 1 mA from the floor of the room. After 24 h, each rat was placed again in the bright chamber. The latency of stepping through the dark part (maximum 600 s) was measured and logged as the index for the passive avoidance behavior [32, 33].

#### 2.6.2. Morris Water Maze (MWM)

Morris water maze apparatus was applied for spatial memory testing. This apparatus includes a black circular pool (height 50 cm and 160 cm in diameter) filled with 30 cm of water (25 ± 2°C). The pool was divided into four quadrants (1, 2, 3, and 4). An invisible platform (280 mm in height and 100 mm in diameter) was located 20 mm below the water at the center of the third quadrant, which remained in the same quadrant throughout the experiment. Each trial was started with the rat being placed in the pool at one of the four cardinal positions around the Pool environment according to a pseudorandom sequence. The topmost duration of the trial was 60 s. Rats were assessed in eight trials (twice from each starting place) for each session for 3 days. On the 4th day, a probe test was done through the platform removal. The rats could swim at will for 60 s. A computer tracking system was used for measuring the time for reaching the platform and spending in the target quadrant [34, 35].

### 2.7. Tissue Preparation

The animals were decapitated and then the whole brain was rapidly dissected, washed with isotonic saline, dried, and then weighed (*n* = 10). Next, it was homogenized immediately to give a 10% (w/v) homogenate in an ice-cold medium containing 50 mmol/l Tris-HCl (pH 7.4) and 300 mmol/l sucrose. The homogenate tissue was centrifuged at 3000 rpm for 10 min. Biochemical analysis (AChE, CAT, GSH, and ferric-reducing antioxidant power) was done after separating the supernatant. All processes were carried out at 4°C. Supernatants were used to determine AChE activity and the level of the antioxidant parameter using a spectrophotometer (Shimadzu UV-mini 1240).

For histopathological examination, the brains were removed and fixed (*n* = 5). Then tissue processing and serial coronal cutting into 8 *µ*m thick (from the bregma, −2.5 mm to −4.5 mm) were performed. The sections were stained with hematoxylin and eosin (H&E) [36] and cresyl violet (CV) [37].

Histopathological lesions (necrosis and gliosis) were scored as previously described [38]. To neuronal count the cortex and the hippocampus, the images were taken from hippocampal regions and cortex. Hence, 45 sections (nine sections from a single animal in each group) were analyzed for each experimental group. Measurement of the area was performed using the capture software (Tucsen, Fuzhou, China). Histological evaluations were performed by pathologists blinded to the experimental groups.

### 2.8. Tissue Cholinesterase Activity Measurement

For the cholinesterase activity assay, the kinetic photometric method was adopted. Cholinesterase can break down butyrilthiocholine to thiocholine which reacted with 5, 5′-dithiobis-2-nitrobenzoate (DTNB), forming the yellow product of 5-thio-2-nitrobenzoate with an absorbance at 412 nm. The rate of absorbance change was regulated to 1 g tissue and it was compared with the references. Cholinesterase activity was calculated using a molar extinction coefficient value of 13.6 mM expressed as U/mg protein. We used a human blood serum sample for positive control provided by healthy volunteers of our lab personnel [39]. The cholinesterase activity was calculated using an extinction coefficient of thiolate dianion of DTNB as a product at 412, which was produced by the enzymatic hydrolysis of acetylthiocholine iodide. In this method, it is not necessary to draw a calibration curve.

### 2.9. Measurement of Malondialdehyde (MDA)

Tissue homogenate (0.5 ml) was diluted to 1 ml using Tris-HCl buffer, then incubated at 37°C for 2 h. Afterward, 1 ml of cold trichloroacetic acid (TCA) was added, vortexed, and centrifuged at 800 × *g* for 10 min. To 1 ml of supernatant was added 1 ml of thiobarbituric acid (TBA) and the reaction mixture was placed in a boiling water bath for 15 minutes. The pink-colored complex was formed whose absorbance was read at 532 nm. The amount of MDA formed (index of lipid peroxidation) was calculated using an extinction coefficient of 1.56 × 10^5^ M^−1^·cm^−1^ for MDA-TBA chromophore, and the results are expressed as nmol/mg protein [40].

### 2.10. Measurement of Catalase Activity

CAT activity was measured based on its capability to decompose hydrogen peroxide (H_2_O_2_) in brain tissue, [41]. As a rule, H_2_O_2_ decomposition can be assessed by a decrease in absorbance at 240 nm. Thus, hydrogen peroxide at a final concentration of 19 mM and 50 mM phosphate buffer (pH 7) was used as a substrate and an alternative substrate in the blank solution, respectively. The reaction was initiated by the addition of H_2_O_2_ and the decrease in the absorbance was evaluated by a spectrophotometer (Pharmacia, Novaspec II, and Biochrom, England) at 240 nm for 30 s. The values were expressed as U/mg protein. To calculate u/mg, the enzyme activity was calculated in U/ml based on changes in the adsorption mixture per minute, then the protein concentration was measured in mg/ml and the activity was divided by the protein concentration of enzyme activity in U/mg. Also, it is not necessary to draw a standard diagram to obtain the activity, and the activity is obtained by using the extinction coefficient related to hydrogen peroxide [41].

### 2.11. Measurement of Ferric-Reducing Antioxidant Power (FRAP) Assay Test

The FRAP value was measured by comparing the change in absorbance at 593 nm in a test reaction mixture containing the mixture (tissue samples with 2,4,6-tri-(2-pyridyl)-s-triazine) with a defined ferrous ion concentration. When reduced to the ferrous form (Feп) in lower pH values, the FeШ-2,4,6-tri-(2-pyridyl)-s-triazine complex produces an intense blue color product with absorption at 593 nm. Practically, conditions that are favorable for complex development are provided in the presence of reductants 0 (antioxidants), which allow color development. A standard solution of ferrous sulfate (Feп100 to 2000 mM) was prepared in distilled water. The FRAP value is expressed as mmol of tissue weight [42].

### 2.12. Measurement of Glutathione (GSH)

GSH measurement was performed by the Ellman procedure. For this purpose, 1 ml of supernatant was taken after sedimentation of 0.5 ml of brain homogenate with 2 ml of 5% TCA. Then, 0.5 ml of Ellman's reagent (0.0198% DTNB in 1% sodium citrate) and 3 ml of phosphate buffer (1 M, pH 8.0) were added. The developed color was observed at 412 nm. A standard curve was drawn using known levels of reduced GSH concentration and described as mg/g of tissue [43].

### 2.13. Statistical Analysis

Data are expressed as the mean ± standard error. Latency data and covered distance during the training days were analyzed using repeated measures within groups, two-way analysis of variance (ANOVA), followed by a Tukey post hoc test to compare between groups [44]. Additionally, Kruskal–Wallis and Mann–Whitney *U* tests were used for histopathological scoring differences between the groups. The statistical comparisons were analyzed through SPSS 26 version [25, 45].

## 3. Result

### 3.1. GC-MS Analysis

The GC-MS analysis of *R. damascena* showed 29 combinations of phytochemicals. The most important substances were furfural, quinic acid, geraniol, and citronellal. Components under 0.1% were removed and combined (Table 1).

### 3.2. Behavioral Scores

#### 3.2.1. Single-Trial Passive Avoidance Test

Average behavioral change scores for (Ctrl), (Sham), (AL), (AL + DRE 500), (AL + DRE 100), (DRE 500), and (DRE 1000) groups were 480, 480, 47.4, 305.9, 373.9, 480, and 480, respectively. There was a significant difference between (AL) group and the control group in retention in the passive avoidance test (*P* < 0.0001). Also, in the AL + DRE 100 group, there was not any significant difference compared to the control group (*P* > 0.05).

#### 3.2.2. Morris Water Maze (MWM)

**Figure 1:**
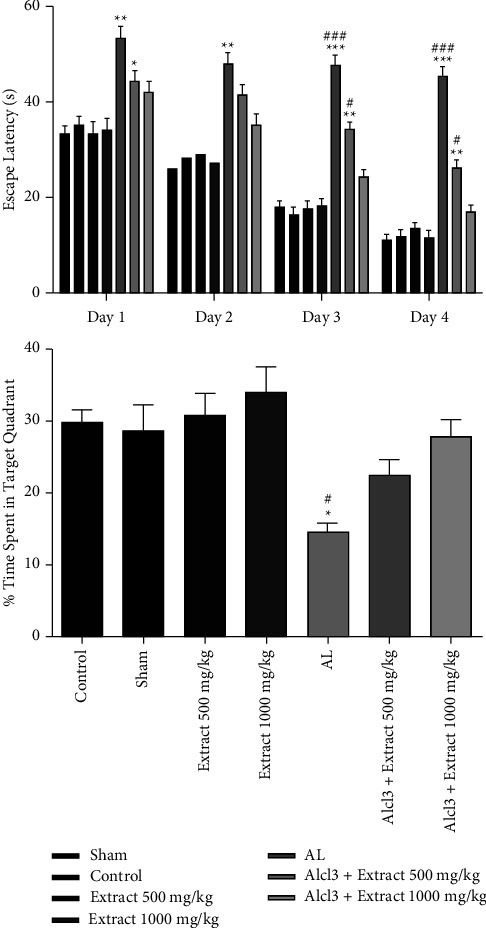
Effect of DRE on escape latency, and the mean percentage of time spent in the target quadrant. Al increased escape latency and *R. damascene* administration significantly reduced the escape latency parameter. ^*∗*^*P* < 0.05, ^*∗∗*^*P* < 0.01, and ^*∗∗∗*^*P* < 0.001 of Al group compared to the control group; ^#^*P* < 0.05 and ^###^*P* < 0.001 compared to Al + extract 1000 mg/kg experimental animal group. During the probe trial, which is an index of spatial memory, it was shown that animals treated with Al + extract 1000 mg/kg had a significant preference for the target quadrant in contrast to the Al group. *P* < 0.01 Al group compared to the control group; *P* < 0.01 Al group compared to the Al + extract 1000 mg/kg group.

*(1) Latency*. There was no difference between groups in terms of latency in the pretest MWT assessment (*P* > 0.05). There was a statistically significant difference between the AL and control groups in escape latency (*P* < 0.01). Escape latency reduction was observed in animals that received the extract (treatment groups). Group AL + DRE 1000 showed no significant escape latency compared to the control group (*P* > 0.05). According to the results, treatment with *R. damascene* reduced the escape latency in the treatment groups compared to the Al group (Figure 1).

*(2) Probe Trial*. The amount of time spent in the target quadrant during the first half of the probe trial session was assessed in the post-test phase. The time spent in the target quarter between the AL and control groups was significantly different. The AL + DRE 1000 group showed improved spatial memory and a significant increase in the time spent in the target quadrant compared to the Al group (*P* < 0.01, Figure 1).

### 3.3. Malondialdehyde (MDA)

The highest level of (MDA) was observed in the AL group. The MDA level decreased in groups AL + DRE 500 and AL + DRE 1000. There was not any significant difference in MDA level between AL + DRE 1000 and the control group (*P* > 0.05) but there was a significant difference between the AL group in comparison to the sham and control groups (*P* < 0.0001, Table 2).

### 3.4. Ferric-Reducing Antioxidant Power (FRAP)

FRAP values in the aluminum group were the lowest while it was increased in groups AL + DRE 500 and AL + DRE 1000. It seems that the negative effects of aluminum are treated with *R. damascene* extract at a dose of 1000 mg/kg. Also, there was not any significant difference in FRAP level in the AL + DRE 1000 group compared to the control group (*P* > 0.05, Table 2).

### 3.5. Glutathione (GSH) and Catalase Activities

The enzyme activities of catalase and GSH were reduced in the AL group compared to the control group (*P* < 0.002). Administration of *R. damascene* significantly increased the catalase activity in the AL + DRE 1000 group. In addition, there was not any significant difference in GSH enzyme activities between the AL + DRE group 1000 and the control group in catalase activity. Regarding the GSH enzyme activities, there was not any remarkable difference in the AL + DRE 1000 group compared to the control group. The best treatment was in the AL + DRE 1000 group in both GSH and catalase activity compared to the control group (*P* > 0.05, Table 2).

### 3.6. Acetylcholinesterase Activity (AChE)

Chronic AL treatment significantly increased brain AChE activity compared to the control group. However, treatment with *R. damascene* significantly attenuated AChE activity (especially in the AL + DRE 1000 group) compared to the control group (*P* > 0.05, Table 2).

### 3.7. Histopathological Examination

**Figure 2:**
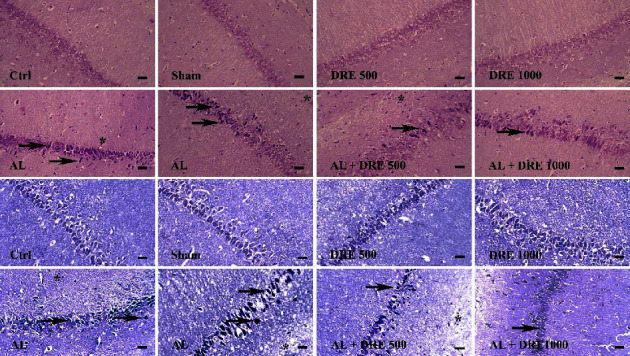
Hippocampus tissue: *Rosa damascena* extracts reduced necrosis and gliosis induced by AlCl_3_ in different regions of the hippocampus. Necrosis and gliosis were not observed in the control, DRE 500 and DRE 1000 groups in the hippocampus, AlCl_3_ group with necrosis (black arrow), and gliosis (star) in the different regions of the hippocampus. In AL + DRE 500, AL + DRE 1000 groups showed less necrosis and gliosis compared to the AlCl_3_ group, ×40 magnifications, H&E, and the Nissl staining. Scale bar = 100 *μ*m.

**Figure 3:**
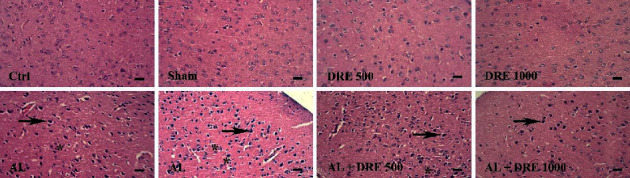
Cortex tissue: *Rosa damascena* extracts (DRE) reduced necrosis and gliosis induced by AL in the cortex. Necrosis and gliosis were not observed in the control, DRE 500 and DRE 1000 groups in the cortex, AL group with necrosis (black arrow), and gliosis (star) in the cortex. In AL + DRE 500, AL + DRE 1000 groups showed less necrosis and gliosis compared to the AL group, ×40 magnifications, and H & E staining. Scale bar = 100 *μ*m.

There was no lesion observed in the brain and hippocampus tissues in the control, sham, DRE 500, and DRE 1000 groups. The brain and hippocampus tissue of the AL group showed the presence of necrosis and gliosis. According to the results, treatment with *R. damascene* reduced the histopathological lesion in the treatment groups. The AL + DRE 1000 group showed significantly reduced necrosis and gliosis compared to the AL group (*P* < 0.002) (Figures 2 and 3; Table 3).

In addition, exposure to AL significantly reduced the number of cells in the cerebral cortex and different regions of the hippocampus compared to the control group (Table 4). *R. damascene* treatment significantly increased the cell numbers in different regions of the hippocampus and cortex compared to the AL-treated group (Table 4).

## 4. Discussion

In this study, it was observed that aluminum is a neurotoxicant and accumulates in different parts of the brain. Al induces oxidative stress and increases MDA and reduces antioxidative enzymes such as SOD, CAT enzyme activities, and TAC levels in the rat brain [46–48]. Our results showed the highest level of MDA was observed in the AL group and the levels of catalase, FRAP, and GSH were reduced in the group AL, our findings are in agreement with previous studies.

Overall, Al-induced oxidative stress in brain tissue and can cause the death of neuronal cells by shifting ROS production. It should be noted that reactive oxygen types (ROS) resulting from a normal aerobic metabolism are potentially harmful. These free radicals are usually removed or disabled by antioxidant groups in vivo. However, since *R. damascene* is a promising source of antioxidants, it was likely to see reduced lesions in the treatment groups after it reduced the free radicals in the tissue. Also, the potential of treatment was investigated by employing DRE at 2 different doses [29].

*R. damascene* is an herbal medicine that is used as a useful and effective treatment against a wide range of diseases and stimulates the nervous system, reduces depression, improves brain function, eliminates fatigue, and prevents plenty of diseases. Antioxidant and hepatoprotective effects of the extract of *R. damascene* have been studied by Taleghani et al. [49]. It seems, according to the properties of the compounds in the *R. damascene* extract such as antioxidant and anti-inflammatory effects and inhibition of AChE activity, it can improve brain function. In this study, the effect of the compounds in the *R. damascene* reduced the adverse effect of aluminum.

Natural Compounds in the *R. damascene* extract have benefic biological effects such as antioxidant and anti-inflammatory activities including dopamine [50], 4H-pyran-4-one,2,3-dihydroxy-3,5-dihydroxy-6-methyl [51], nonadecane [52], 2,3-dihydroxypropanal [53], ethenone [54], tert-butyl [4-(hydroxymethyl) cyclohexyl] carbamate [55], 4H-pyran-4-one,2,3-dihydroxy-3,5-dihydroxy-6-methyl, myristylamine [56], citronellal [57, 58], benzaldehyde [59], geraniol [60], and citronellol [61, 62]. Furfural is an organic compound that was found (28.14%) in the *R. damascene* extract and has benefic biological effects such as antioxidant, antihypoxia, and hepatoprotective activities [63, 64]. Geraniol is an approved natural antioxidant with plenty of benefits in the regulation of the body's homeostasis [65]. Similarly, citronellol is a strong antioxidant, which was shown to be effective in inhibiting certain enzymes [61].

Our results show that the administration of *R. damascene* extract increased catalase and glutathione levels in the treatment groups. Hence, regarding the protective and antioxidant effects of *R. damascene* extract, the best form of efficacy was exhibited in a group of (AL + DRE 1000). We also found the highest level of MDA in the AL group and the enzyme activities of CAT, FRAP, and GSH reduced in the group AL, confirming earlier studies.

Acetylcholine is an important neurotransmitter in the memory and learning process. The behavior and performance deficits in AD are due to the inability the transmission of nerve impulses in the cholinergic synapses [66]. AChE plays a role in cholinergic transmission. In addition, cholinesterases may also play a role during morphogenesis and in the onset of neurodegenerative diseases [67]. The observed elevated activity of AChE is described as the direct effect of aluminum [68]. Aluminum is a strong cholinotoxin that has a biphasic effect on acetylcholinesterase activity with a primary increase in the activity of this enzyme. This biphasic effect is ascribed to the gradual agglomeration of Al in the brain and this is the reason for increasing AChE in the rat [69].

Chlorogenic acids are a family of polyphenolic compounds, which is esters such as quinic acid that are found in *R. damascene* extract. Chlorogenic acids are potent antioxidants [70] and inhibited acetylcholinesterase activity in the frontal cortex and hippocampus [71]. Also, quinic acid has been characterized as a prometabolite that leads to the induction of efficacious levels of tryptophan and nicotinamide as antioxidants [72]. The results of our study showed aluminum chloride administration increased AChE activity and *R. Damascene* extract attenuated AChE activity.

Because of the presence of this material and the antioxidant characteristics of the plant, it can be used as a treatment and prevention of brain illnesses such as AD. According to the results of behavioral tests, it was observed that the aluminum group had a significant difference from other groups. The results of this study are consistent with the findings of the study by Burimau, who concluded that aluminum reduced the behavior scores of the Wistar rats [73]. In another study, Azui found gavage administration of aluminum in mice reduced their vigilance [74]. Moreover, Goat and Caorah also found that the intake of AlCl_3_ in male mice causes neuronal damage and behavioral changes [75].

Al administration showed a decrease in spatial memory and notably increased the time needed to overtake the hidden platform. Abulfadl et al. [48] investigated the exposure to aluminum significantly reduced memory in the MWM test and AlCl_3_-induced neurotoxicity in rats. An earlier study reported aluminum administration had a neurodegeneration effect resulting in learning deficits [1, 76]. The results of these studies are consistent with our study.

In this study, *R. damascene* extract improved significantly the latency periods of a passive avoidance response in the treatment groups. Also, *R. damascene* treatment during aluminum exposure reduced the time to reach the hidden platform, which this reduction was more in Al + DRE 1000 group. On the probe day, the time spent on the target quadrant was notably reduced in the AL group compared to other groups. It seemed that the negative effects of aluminum-receiving groups were downregulated with *R. damascene* extract, and both treatment groups, which received 500 and 1000 DRE, respectively, did not show significant differences compared to the control group. Our results are in agreement with the previous study [77] that reported *R. damascene* extract administration improved learning and memory by using Morris Water Maze (MWM) performance and the passive avoidance test in treated rats.

It was indicated that the toxic effects of Al on mice's brain confirm the damage in the hippocampus and cortex, including necrosis, gliosis, and neurofibrillary degeneration due to the accumulation of Al in these regions [78]. These results are in agreement with our study. Similarly, histopathology findings of the brain and hippocampus confirmed the protective activity of *R. damascene* extract against aluminum-induced brain and hippocampus damage, as is evident by the reduction of brain and hippocampus lesions such as gliosis and necrosis in treatment groups, especially in AL + DRE 1000 group.

Overall, it seemed that the extract was quite successful at reducing the Al-induced lesions in brain tissue. These findings are in line with each other, which suggest that can be used as a potent natural substance for fighting with AD. DRE showed than has wide effects on multiple pathways, and can effectively upregulate the functions of the brain such as remembering.

## 5. Conclusion

In summary, this study demonstrated that Al results in toxic effects, inducing oxidative stress, increasing AChE, reduced behavior scores, and histopathological changes in brain tissue. Also, *R. damascene* extract in a dose of 1000 mg/kg could improve significantly the levels of acetylcholinesterase enzymes and the rate of stress indicators, improving the level of consciousness and brain function. Therefore, *R. damascene* extract can be used as an effective treatment agent for preventing complications from Al intoxication.

## Figures and Tables

**Table 1:** Compounds that were identified in the aquatic extract of *Rosa damascena* by GC-MS.

Chemical constituents	Retention time (minutes)	Peak area (%)	Molecular weight (g/mol)	Molecular formula
Furfural	18.903	28.14	96.085	C_5_H_4_O_2_
Furfuryl alcohol	5.361	1.25	98.101	C_5_H_6_O_2_
Phenethyl alcohol	13.533	5.48	122.17	C_8_H_10_O
Citronellol	16.179	6.35	156.27	C_10_H_20_O
Nonadecane	36.709	2.98	268.529	C_19_H_40_
Heneicosane	41.709	1.78	296.583	C_21_H_44_
Eicosane	46.757	0.77	282.556	C_20_H_42_
Phthalic acid	49.936	1.41	166.132	C_8_H_6_O_4_
Clionasterol	59.375	3.59	414.718	C_29_H_50_O
Methoxyphenylacetone	11.082	0.17	164.204	C_10_H_12_O_2_
Quinic acid	34.342	22.90	192.167	C_7_H_12_O_6_
Hexacosanoic acid	46.932	0.11	396.7	C_26_H_52_O_2_
Ethanone	48.416	2.42	313.4	C_22_H_19_NO
Octadiene	50.118	1.12	110.2	C_8_H_14_
--1-(4-Chlorophenyl)	4.367	0.18	191.658	C_11_H_10_ClN
2,3-Dihydroxypropanal	10.368	0.32	180.156	C_6_H_12_O_6_
Dopamine	12.559	1.46	153.181	C_8_H_11_NO_2_
Adipic acid	15.465	0.16	146.142	C_6_H_10_O_4_
Geraniol	22.236	9.39	154.25	C_10_H_18_O
Propylamine	29.903	1.55	59.112	C_3_H_9_N
Myristyl amine	38.950	0.34	213.409	C_14_H_31_ N
Citronellal	47.128	0.29	154.253	C_10_H_18_O
Benzaldehyde	55.292	0.28	106.124	C_7_H_6_O
Other compounds		7.59		

**Table 2:** Effect of *R. damascene* extract (DRE) on aluminum-induced oxidative stress parameters and AChE activity in rat brain.

Group	FRAP (mmol/g protein)	MDA (nmol/mg protein)	GSH (*µ*mol/g tissue)	Catalase (U/mg protein)	AChE (U/mg protein)
Control	302.62 ± 21.76^c^	24.11 ± 2.23^a^	1.99 ± 0.233^b^	0.830 ± 0.095^b^	1.176 ± 0.173^a^
Sham	302.45 ± 26.54^c^	25.8 ± 2.25^a^	1.78 ± 0.212^b^	0.929 ± 0.081^b^	1.20 ± 0.228^a^
DRE 500 mg/kg	309.91 ± 25.47^c^	24.53 ± 1.91^a^	1.99 ± 0.139^b^	0.785 ± 0.108^b^	1.71 ± 0.309^ab^
DRE 1000 mg/kg	314.90.8 ± 39.98^c^	25.43 ± 1.82^a^	2.25 ± 0.230^b^	0.878 ± 0.089^b^	1.80 ± 0.342^ab^
AL	197.88 ± 21.76^a^	58.82 ± 5.21^c^	0.958 ± 0.158^a^	0.309 ± 0.062^a^	3.62 ± 0.348^c^
Al + DRE 500 mg/kg	210.0 ± 25.10^a,b^	49.46 ± 4.03^b^	1.104 ± 0.130^a^	0.473 ± 0.106^a^	2.44 ± 0.319^b^
Al + DRE 1000 mg/kg	276.9 ± 19.88^b^	28.63 ± 2.58^a^	1.69 ± 0.109^b^	0.860 ± 0.051^b^	1.56 ± 0.303^ab^

**Table 3:** Effect of *R. damascene* extract(DRE) on aluminum-induced histopathological changes on the cortex and hippocampus of rats.

Parameters	Cortex		Hippocampus	
	Necrosis	Gliosis	Necrosis	Gliosis
Control	0.1 ± 0.10	0 ± 0	0.1 ± 0.10	0 ± 0
Sham	0.1 ± 0.10	0 ± 0	0.1 ± 0.10	0 ± 0
DRE 500	0.1 ± 0.10	0 ± 0	0.1 ± 0.10	0 ± 0
DRE 1000	0.1 ± 0.10	0 ± 0	0.1 ± 0.10	0 ± 0
AL	2 ± 0.26^a^	1.2 ± 0.25^a^	1.5 ± 0.22^a^	1.1 ± 0.23^a^
AL + DRE 500	1.7 ± 0.21^e^	0.8 ± 0.20^e^	1.3 ± 0.15^e^	0.6 ± 0.16^e^
AL + DRE 1000	1 ± 0.26^b,d^	0.4 ± 0.16^b,d^	0.7 ± 0.15^b,c,d^	0.3 ± 015^b^
*P* value^*∗*^	0.000	0.000	0.000	0.000

Values are expressed as means ± standard error (SE), for each group. ^*∗*^Asymptotic significances differences of cortex and hippocampus lesions between groups (*P* ≤ 0.0001; Kruskal–Wallis test). ^a^Statistically significant differences between (control, sham, DRE 500, and DRE 1000) and AL (Mann–Whitney *U* test; *P* ≤ 0.001). ^b^Statistically significant differences between AL and AL + DRE 1000 (Mann–Whitney *U* test; *P* ≤ 0.02). ^c^Statistically significant differences between AL + DRE 500 and AL + DRE 1000 (Mann–Whitney *U* test; *P* ≤ 0.002). ^d^Statistically significant differences between (control, sham, DRE 500, and DRE 1000) and AL + DRE 1000 (Mann–Whitney *U* test; *P* ≤ 0.03). ^e^Statistically significant differences between (control, sham, DRE 500, and DRE 1000) and AL + DRE 500 (Mann–Whitney *U* test; *P* ≤ 0.004).

**Table 4:** Effect of *R. damascene* extract on aluminum-induced neuronal count on the cortex and hippocampus of rats.

	CA1	CA23	CA4	DG	Cortex
Control	210.2 ± 3.68^a^	191.1 ± 5.38^a^	86.9 ± 2.12^a^	195.4 ± 2.68^a^	218.4 ± 3.26^a^
Sham	211 ± 3.53^a^	188.8 ± 5.37^a^	86.4 ± 2.33^a^	191.2 ± 2.83^a^	214.6 ± 4.08^a^
DRE 500	211.5±4^a^	188.7 ± 3.66^a^	87.3 ± 1.33^a^	195.5 ± 3.1^a^	220.6 ± 2.89^a^
DRE 1000	214.1 ± 3.49^a^	195.7 ± 3.66^a^	93.2 ± 1.74^a^	198 ± 2.77^a^	222.4 ± 3.67^a^
AL	121.9 ± 4.89^c^	99.9 ± 4.01^c^	49.5 ± 1.59^d^	144 ± 3.83^c^	154.2 ± 5.74^d^
AL + DRE 500	170.5 ± 3.65^b^	147.1 ± 3.7^b^	66.1 ± 1.23^c^	163.9 ± 4.11^b^	185 ± 5.43^c^
AL + DRE 1000	178.1 ± 3.67^b^	154.9 ± 3.74^b^	73.6 ± 1.30^b^	171.6 ± 4.52^b^	200.4 ± 5.49^b^

## Data Availability

Data are available from the corresponding author upn a reasonable request.

## Abbreviations

Al: Aluminum
DRE: Aqueous *R. damascena* extract
AChE: Acetylcholinesterase
CAT: Catalase
AD: Alzheimer's disease
TAC: Total antioxidant capacity
SOD: Superoxide dismutase
MDA: Malondialdehyde
GSH: Glutathione.
